# Infection risk of peripheral intravenous catheters: meta-synthesis of 18 prospective studies with 14,606 catheters

**DOI:** 10.1186/s13756-025-01645-z

**Published:** 2025-10-27

**Authors:** Claire M Rickard, Jessica Schults, Gabor Mihala, Emily Larsen, Nicole Marsh, Naomi Runnegar, Tricia Kleidon, Amanda J Ullman, Samantha Keogh, Daner Ball, Amanda Corley, Simon Bugden, Gillian Ray-Barruel

**Affiliations:** 1 Metro North Health, Herston Infectious Diseases Institute, Herston, QLD 4006 Australia; 2 School of Nursing, Midwifery and Social Work, The University of Queensland Centre for Clinical Research, Herston, QLD 4006 Australia; 3 Alliance for Vascular Access Teaching and Research, Schools of Nursing and Midwifery and Pharmacy and Medical Sciences, Griffith University, Southport, QLD 4215 Australia; 4 Nursing and Midwifery Research Centre, Royal Brisbane and Women’s Hospital, Herston, QLD 4029 Australia; 5 Centre for Health Services Research, The University of Queensland, Brisbane, Australia; 6 Australasian Kidney Trials Network, The University of Queensland, Brisbane, Australia; 7 Princess Alexandra Hospital, Brisbane, QLD Australia; 8 The University of Queensland, Brisbane, Australia; 9 Children’s Health Queensland Hospital and Health Service, Queensland Children’s Hospital, Brisbane, QLD Australia; 10 Children’s Health Research Centre, University of Queensland, Brisbane, Australia; 11 School of Nursing and Centre for Healthcare Transformation, Queensland University of Technology, Brisbane Queensland, 4059 Australia; 12 Metro North Health Service, Brisbane, QLD Australia

**Keywords:** Peripheral intravenous catheter, Vascular access devices, Bloodstream infection, Local infection, Staphylococcus aureus, Meta-synthesis, Epidemiology

## Abstract

**Background:**

To quantify the incidence of peripheral intravenous catheter (PIVC) infections and to describe the influence of clinical characteristics, including dwell time, on risk.

**Methods:**

Meta-synthesis of 18 prospective studies (16 randomized controlled trials and two prospective cohort studies) reporting PIVC infections. In total, 14,606 PIVCs (50,096 device-days) were studied from insertion to removal in seven Australian government hospitals. PIVC care was provided by clinical staff with daily follow up by research nurses. We calculated incidences and rates of local infection (without bloodstream infection [BSI]) and PIVC-associated bloodstream infection (i.e., primary BSI) using the National Healthcare Safety Network criteria. The hazard function was assessed by fitting a parametric survival model. PIVC-associated BSI was further categorized as PIVC-related BSI and/or *Staphylococcus aureus* BSI. Case study methodology explored characteristics of PIVC-associated BSI, and life tables explored the hazard function of PIVC-associated BSI over dwell time.

**Results:**

Of 14,606 PIVCs (dwell 0–42 days), there were five local infections (0.034%; 0.100/1,000 device-days) and six PIVC-associated BSI (0.041%; 0.120/1,000 device-days), of which four were PIVC-related and one was *S. aureus* BSI. PIVC-associated BSI involved *Enterobacter cloacae* (*n* = 3 including one co-infection with *Citrobacter braakii*), *Proteus mirabilis* (*n* = 1), *Pseudomonas aeruginosa* (*n* = 1) and *S. aureus* (*n* = 1; *S. aureus* BSI incidence 0.007% catheters or 0.020/1000 device-days). PIVC-associated BSI cases commonly featured: males > 60 years with difficult intravenous access, delayed removal of idle or symptomatic PIVCs, cancer diagnoses, invasive gastrointestinal drains/procedures, insertion site complications, and forearm placement. PIVC-associated BSI daily hazard was constant over time with zero to 0.03% on Days 1 to 5 (*n* = 11,491), 0.06% to 0.10% on Days 6 and 7 (*n* = 2,571), and zero on Days 8 to 42 (*n* = 544).

**Conclusions:**

Infection incidence is very low but remains a serious risk, mainly for complex patients. Gram-negative organisms may now be predominant in Australia. Infection surveillance should be risk-adjusted and prevention efforts to improve both insertion and post-insertion management targeted at high-risk groups. While overall intravenous therapy (exposure) should be minimised, daily risk per PIVC appears constant for at least 5 days.

**Supplementary Information:**

The online version contains supplementary material available at 10.1186/s13756-025-01645-z.

## Background

Peripheral intravenous catheters (PIVCs) can be the source of primary bloodstream infections (BSIs) involving Gram-positive or Gram-negative bacteria, fungi, or polymicrobial organisms, all of which have serious mortality and morbidity risks [1–3]. PIVC-associated BSIs constitute 13% to 23% of all intravascular catheter-associated BSIs, and 4% to 6% of all healthcare-associated BSIs [1, 4–6]. As most hospitalised patients receive a PIVC, a substantial number of people are at risk of these infections each year [1, 7].

Currently, precise risk estimates per catheter are precluded by large numbers of undocumented PIVC insertions in medical records [7], surveillance databases that lack denominators of all PIVCs used in the facility, PIVC days or risk stratification, and few studies with follow up after PIVC removal/hospital discharge, low rates of microbiological investigation of suspected infections, and the absence of publicly available surveillance data. Recent systematic reviews of PIVC-associated BSI reported a six-fold difference in incidence per catheter of 0.028% (95% confidence interval [CI] 0.01–0.08) [8] and 0.18% (range 0–2.2%) [5]). An older systematic review reported PIVC-related BSI (a more stringent diagnostic definition requiring microbiological evidence of the PIVC as the source) in the middle of this range at 0.1% (95% CI 0.1–0.2) of PIVCs [9].

PIVC-associated BSI commonly involves *Staphylococcus aureus (S. aureus BSI i.e.*,* SABSI)*, which significantly increases risk of further complications (e.g., infective endocarditis) and 30-day mortality [1, 2]. SABSI has been reported to occur in Australia at 0.76 per 10,000 hospital-days [10], and a systematic review indicates the proportion of these that are also PIVC-associated BSI is 19% (range, 8–35%) [5]. Local (soft tissue) PIVC infections can also occur and may indicate early or non-confirmed PIVC-associated BSIs. Incidence data on PIVC local infections is less commonly available, complicated by inconsistent definitions, which may be limited to phlebitis (which can be infective or alternatively have chemical or mechanical aetiology) [5]. Local infection was quantified in a recent systematic review at 0.15% PIVCs (95% CI 0.047–0.479; 30 studies) [8].

In addition to incidence uncertainty, risk factors for PIVC-associated BSI are understudied, limiting the ability to risk-stratify focussed monitoring and prevention efforts in these patients. The limited number of published epidemiologic studies on risk factors reflects the difficulty of studying a low incidence condition, with studies predominantly retrospective, single-centre cohort designs. Factors significantly associated with PIVC-associated BSI in multivariable analyses have been older age, showering with a PIVC, and increased severity of illness [3],. PIVC-*related* BSI risk factors have been reported as intensive care unit (ICU) admission, male sex, large catheter gauge (≤ 16G) and hospitalisation during the 2021 COVID-19 pandemic [11]. Local infection risk factors have been reported as medical (not surgical) admission, “emergency room or ward nurse” inserters (not PIVC therapists), and continuous infusions (not open systems) [12] and insertion over a joint (cubital fossa or wrist) [11]. Logically, more overall PIVC-days increases infection risk, but there is ongoing uncertainty about whether risk increases linearly over time, above and beyond the ‘per-day’ risk [3, 12].

Recognising the substantial risk posed by PIVCs across the health system, Australia implemented a national Clinical Care Standard in 2021 [13] and the World Health Organization recently released global guidelines [14]. It is expected that as compliance with these documents increases, PIVC infections will reduce. In anticipation of this improvement and to support their implementation, we aimed to synthesize the rate and risks of infections in PIVCs observed in multiple research studies undertaken by our network. Our aims were to: (1) quantify PIVC infection risk (likelihood of infection) in acute care settings, (2) describe common clinical characteristics of patients and/or devices with PIVC infections, and (3) compare per-day infection risk.

## Methods

### Design

Meta-synthesis of infection data from 18 prospective PIVC research studies (16 RCTs and two observational studies), undertaken by clinician investigators affiliated with the Alliance for Vascular Access Teaching and Research (AVATAR) (www.avatargroup.org.au).

### Research questions

1. What is the risk of local infections and BSIs developing per PIVC?
2. What are the clinical scenarios surrounding PIVC-associated BSI?
3. Does per-day PIVC-associated BSI risk increase linearly over time?

### Inclusion criteria

We included AVATAR-affiliated studies that (1) prospectively enrolled PIVCs at insertion and followed them until removal, (2) reported infection outcomes, and (3) measured dwell time. This approach was taken as there was a large database available with high-quality data. Although patients and hospitals varied, the overarching methodologies for follow-up and variable definitions were largely standardised. The sample size was determined by the number of studies available.

### Overview of primary studies

The 18 studies were published between 2007 and 2023 with a combined 14,606 PIVCs representing 50,096 device-days [15–33]. Studies were performed in eight government-run, university-affiliated hospitals in metropolitan (*n* = 3), or regional (*n* = 4) areas in Australia. Participants were hospital inpatients, including one hospital-in-the-home (inpatients but cared for by hospital staff in their home) study [15] and two paediatric studies [26, 27]. Sixteen studies were RCTs of varied clinical practices or medical devices: e.g., dressings and securements [18, 19, 22, 24, 26, 31], removal policies [15–17], levels of inserter [23], flushing protocols [20, 27, 28], connectors [30, 32, 33], or catheters [30]. Most RCTs were comparative effectiveness trials comparing existing approaches to care and so both intervention and control groups were included in this meta-analysis, with the exception of one RCT for which we excluded the arm with midline catheters [34]. Two studies were observational [25, 29]. This meta-synthesis constitutes an inception cohort, with patients recruited and followed from PIVC insertion (most inserters were bedside nurses and junior doctors). Post-insertion care and decisions about removal were made by the usual clinical staff (not researchers or IV teams).

### Data collection

Data were collected prospectively by research nurses using direct patient assessment, questioning of patients/parents/staff, and reviewing hospital records. Patients were followed until 48 h post PIVC removal except for one emergency department study [19] in which follow-up was for 48 h post-insertion. Research nurses were trained and supervised by a study manager, with data recorded on electronic case report forms, separate to hospital charts and not visible to clinical staff. Infection endpoints were allocated by a blinded infectious diseases physician using definitions consistent with the 2024 NHSN CLABSI criteria for PIVC-associated BSI (modified by substituting PIVCs for central venous lines), VASC criteria for PIVC site infections, and others as relevant for secondary BSIs [35]. Microbiological specimens were ordered by treating staff only for clinical reasons, not routinely.

### Data extraction

We extracted and pooled the following variables: number of PIVCs studied, hours of PIVC dwell, insertion site assessments (e.g., pain, erythema), microbiology results, clinical and demographic details.

### Primary endpoints

There were two co-primary endpoints: (1) local PIVC infection defined using the NHSN *Cardiovascular System VASC-Arterial or Venous Infection* (CVS-VASC) criteria (these patients do not have a BSI), and (2) PIVC-associated BSI using NHSN primary BSI criteria (see supplementary material) [35]. In short, PIVC-associated BSI patients had a PIVC in situ within the previous 48 h, and no other known source of the primary BSI.

### Secondary endpoints

PIVC-associated BSIs were further categorised as:

1. PIVC-related BSI: Meeting elements of both CVS-VASC and Primary BSI definitions as outlined above, to identify infections with stringent evidence of the PIVC as the source.
2. PIVC-associated SABSI: Primary BSIs involving *S. aureus.*

The primary and secondary endpoints were ascertained in the primary studies by a blinded infectious disease physician. Case study data for patients with primary BSI were each reviewed by two of three raters (CMR, EL, DB) and reviewed by an infectious disease physician (NR). Case study variables were: (1) patient (e.g., demographics, diagnosis and comorbidities), (2) PIVC (e.g., insertion procedure, management, ‘idle’ status (unused > 24 h), (3) insertion site assessments (e.g., reddened, painful), (4) PIVC removal (e.g., reasons for and clinical context), and (5) the PIVC-associated BSI episode (e.g., symptoms, treatment, and response). Dwell time at the time of infection was drawn from the primary studies.

### Data analysis

Infection outcomes were presented as incidence per 100 PIVCs (i.e., percentage) and incidence rate per 1000 PIVC-days. Microorganisms were described qualitatively. Case study details were synthesized and described narratively with a focus on similarities between cases. Dwell hours were converted to the day of PIVC removal, with removal < 1–24 h after PIVC insertion coded as Day 1. Five PIVCs categorised as ‘19 days or longer’ dwell in one primary study were analysed as 19 days of dwell for this study. Incidence rates were calculated using Poisson regression (including the natural logarithm of dwell time). Outcomes were assumed to occur at the end of the intervals (days). A life table was generated to display the interval-specific (day-specific) hazard of primary BSI with 95% confidence intervals (CI). The hazard is the probability that a PIVC had a BSI event on a specific day and is therefore conditional on ‘survival’ until the given day. The Nelson-Aalen cumulative hazard and the smoothed hazard function were plotted over time. The shape of the hazard function was assessed by fitting exponential and Weibull parametric survival models. The exponential survival distribution represents hazard level over time, while the Weibull distribution can model both decreasing and increasing risks, depending on the shape parameter *p* (decreasing hazard at *p* < 1, increasing at *p* >1) [36]. Models were compared using Bayesian Information Criterion (BIC); a BIC value closer to zero by more than 2 indicated better model fit [37].

## Results

The 14,606 PIVCs were inserted by general medical and nursing staff, and a smaller number by advanced nurse inserters; ultrasound use was rare (Table 1). Around 18% were inserted in the emergency department and < 1% pre-hospital. Pre-insertion skin decontamination was usually 2% chlorhexidine gluconate in alcohol and sterile transparent dressings were mainly used. About 60% of patients were cared for under a routine 72-h or 72–96-hour removal policy, and the remainder under a clinically indicated removal policy (remove if PIVC no longer needed, not functional, or complications develop). Infection prevention nurse specialists and physicians were present in each hospital to oversee policy and to perform surveillance. The larger metropolitan hospitals had small numbers of specialist vascular access nurses to support institution-wide training, policy, auditing, and insertion of mostly central venous catheters and some PIVCs in difficult intravenous access patients.

**Table 1:** Patient, PIVC and study characteristics for 18 prospective studies

1st author, year Design, *N* = PIVCs	Patients	Insertion	Who inserted	Standard practice	Removal policy
Van Donk [15] RCT, *N* = 316	Adult ED patients discharged to home IV antibiotic service	Hand 19% Cubital fossa 11% Forearm 52% Cephalic fossa/Wrist 15%	Medical doctor 11% Nurse 79% Unknown 10%	70% IPA swab or 70% IPA-CHG swab skin prep.	72–96 h removal: *n* = 161 End of treatment or complications: *n* = 155
Rickard [16] RCT, *N* = 603	Adults Medical 71% Surgical 29% Multiple comorbidities 84% Any infection at baseline 51%	Hand 65% Forearm 18% Cubital fossa 15% Other 2% Ward insertion 67% Emergency insertion 24%	Medical doctor 80% Ward nurse 20%	Alcoholic CHG skin prep. Polyurethane PIVCs Transparent dressings	72 h removal: *n* = 323 End of treatment or complications: *n* = 280
Rickard [17] RCT, *N* = 5907	Adults Medical 19% Surgical 81% Wound infection at baseline 15%	Med-surg ward insertion76% Operating/radiology suite insertion 13% Emergency insertion 10%	Advanced nurse inserter 40% Clinical medical/nursing staff 60% Paramedic < 1%	IPA-CHG skin prep. BD Insyte Autoguard 25–30 mm Non-bordered transparent dressing	72–96 h removal: *n* = 3,215 End of treatment or complications: *n* = 2,692
Marsh [18] RCT, *N* = 85	Adults Medical 47% Surgical 51% Oncology 2% Any infection at baseline 44%	Multiple attempts 14% Forearm 69% Wrist 19% Hand 8% Cubital fossa 4%	Hospital IV Insertion Team 100%	IPA-CHG skin prep. BD Insyte Autoguard 25–30 mm Non-bordered or bordered transparent polyurethane dressing, ± securement device or skin glue	72 h removal
Bugden [19] RCT, *N* = 369	Adult ED patients On antibiotics 33%	Cubital fossa 57% Hand 28% Forearm 16%	Medical doctor 70% Nurse/Other 30%	Alcoholic CHG skin prep. BD Insyte Autoguard 25–30 mm Bordered transparent polyurethane dressing 51%, ± skin glue 59%	72 h removal
Keogh [20] RCT, *N* = 160	Adults Medical 59% Surgical 41% Any infection at baseline 27% Multiple comorbidities 67% Obese 11%	Multiple attempts 20% Forearm 37% Wrist 24% Hand 24% Cubital fossa 14% Upper arm < 1%	Advanced nurse inserter 45% Medical doctor 43% Bedside nurse 9% Other/unknown 4%	IPA-CHG skin prep. BD Insyte Autoguard blood-control 25–30 mm	72 h removal
Marsh 25 Prospective cohort, *N* = 1578	Adults Surgical 67% Any infection at baseline 11% Multiple comorbidities 46% Obese 33%	Multiple attempts 23% Ward 35% Operating theatre 30% Emergency department 22% Pre-hospital 6% Hand 37% Cubital fossa 27% Forearm 24% Wrist 12%	Medical doctor 83% Nurse 11% Paramedic 6%	IPA-CHG skin prep. BD Insyte Autoguard blood control 25–30 mm Simple transparent or bordered transparent dressing 34% Adhesive gauze 13%	72 h removal
Marsh [23] RCT, *N* = 119	Adults Medical 27% Surgical 73% Any infection at baseline 32% Any comorbidities 82% Obese 12%	Forearm 58% Wrist 19% Hand 14% Cubital fossa 6% Upper arm < 1% Foot < 1% Multiple attempts 24%	Advanced nurse inserter 58% Bedside nurse 6% Medical doctor 36%	IPA-CHG skin prep. BD Insyte Autoguard blood-control 25–30 mm Simple transparent dressing 3% Bordered transparent dressing 94%	72 h removal
Marsh [24] RCT, *N* = 300	Adults Medical 28% Surgical 72% Any infection at baseline 27% Multiple comorbidities 77% Obese 14%	Multiple attempts 15% Forearm 73% Wrist 18% Hand 7% Upper arm 1% Cubital fossa < 1% Foot < 1% Ward insertion 99% Operating theatre insertion < 1%	Advanced nurse inserter 99% Medical doctor 1%	IPA-CHG skin prep. BD Insyte Autoguard blood-control 25–30 mm Bordered transparent dressing 50% Integrated Securement Device 50%	72 h removal
Rickard [22] RCT, *N* = 1709	Adults Medical 51% Surgical 44% Cancer 5% Any infection at baseline 19% Obese 14%	Multiple attempts 20% Forearm 68% Wrist 15% Hand 7% Cubital fossa 5% Upper arm 5%	Advanced nurse inserter 88% Bedside nurse 7% Medical doctor 5%	IPA-CHG skin prep. BD Insyte Autoguard 25–30 mm, or BBraun Introcan Safety 3 with short extension BD Smart-Site or MaxPlus Clear Needle-free valve Non-bordered or bordered transparent polyurethane dressing, ± securement device or skin glue	72–96 h removal: *n* = 663 End of treatment or complications: *n* = 1,046
Kleidon [26] RCT, *N* = 319	Paediatrics Medical 36% Surgical 64% Any infection at baseline 33% Multiple comorbidities 17% Obese 9%	Multiple attempts 36% Forearm 45% Hand 26% Cubital fossa 12% Wrist 8% Foot 6% Upper arm 2% Unknown/Other < 1% Ward insertion 38% Operating theatre insertion 31% Procedure Room insertion 28% Emergency insertion 2% ICU/Other insertion 1%	Medical doctor 57% Advanced nurse inserter 41% Bedside nurse < 1% Other/Unknown 1%	2% CHG in 70% IPA swab skin prep. BD Insyte Autoguard 25–30 mm Bordered transparent dressing 33% Bordered transparent dressing with tissue adhesive 34% Integrated securement dressing 34%	Clinically indicated
Keogh [20] RCT, *N* = 619	Adults Medical 45% Surgical 54% Other < 2% Any infection at baseline 9% Multiple comorbidities 74% Obese 4%	Multiple attempts 14% Unknown 44% Forearm 28% Wrist 14% Hand 33% Cubital fossa 24% Foot < 1% Upper arm < 1% Emergency insertion 21% Ward insertion 42% Theatre insertion 14%	Bedside nurse 17% Medical doctor 45% Other/Unknown 37% Advanced nurse inserter < 1%	2% CHG in 70% IPA swab skin prep. BD Insyte Autoguard blood-control 25–30 mm Bordered transparent dressings	72 h removal
Kleidon [26] RCT, *N* = 55	Paediatrics Medical 55% Surgical 40% Other 5% Any infection at baseline 45% Multiple comorbidities 16% “Unhealthy” weight 27%	Multiple attempts 18% Forearm 11% Wrist 4% Hand 45% Cubital fossa 38% Foot 2% Emergency insertion 49% Ward insertion 29% Operating theatre insertion16%	Medical doctor 82% Bedside nurse 11% Other/Unknown 7%	2% CHG in 70% IPA swab skin prep. BD Insyte Autoguard 25–30 mm	Clinically indicated
Larsen [29] Prospective cohort, *N* = 396	Adults Oncology 61% Haematology 39% Any infection at baseline 35% Any comorbidities 77%	Multiple attempts 24% Unknown 7% Forearm 48% Cubital fossa 26% Wrist 13% Hand 8% Upper arm 1% Ward 50% Emergency/Ambulance 25% Outpatient dept 15% Radiology/Procedure room 3% Operating theatre 2%	Bedside nurse 66% Medical doctor 23% Paramedic 4% Other/Unknown 6%	2% CHG in 70% IPA swab skin prep. BD Insyte Autoguard blood-control 25–30 mm Bordered transparent dressings	72 h removal
Rickard [30] RCT, *N* = 1759	Adults Medical 27% Surgical 68% Other 5% Any infection at baseline 15% Any comorbidities 86%	Posterior lower forearm 43% Anterior upper forearm 19% Wrist 12% Cubital fossa 10% Hand 7% Posterior upper forearm 5% Anterior upper arm 2% Other 2% Emergency 9% Ward 90% Other 1%	Medical Doctor 3% Advanced nurse inserter 48% Registered Nurse 48%	B Braun Introcan Safety 3 Catheter BD Connecta 10 cm extension set BD SmartSite needleless connector	72 h removal
Marsh [32] RCT, *N* = 200	Adults Medical 21% Surgical 77% Cancer 2% Infection at baseline 64% Any comorbidities 88%	Multiple attempts 25% Forearm 85% Upper arm 10% Wrist/hand 5% Cubital fossa 1% Ward 100% Theatre < 1%	Research nurse 84% Registered nurse 2% Vascular access service 10% Doctor 4% Other < 1%	BD SmartSite needleless connector BD Insyte Autoguard Blood Control (non-winged) catheters BD Connecta 10 cm extension set 3 M SoluPrep Reynard IPA prep pad.	72–96 h removal
Marsh [34] RCT, *N* = 69 PIVCs (midline catheters excluded)	Adults (PIVC group only) Emergency surgical 58% Planned surgical 23% Emergency medical 16% Planned medical 2% Other 1% Male 52% Infection at recruitment 54% ≥ 3 comorbidities 68% Overweight/obese 68%	Multiple attempts 29% Upper forearm 28% Lower forearm 21% Wrist 21% Upper arm 16% Hand 7% Cubital fossa 7% Ultrasound insertion 12%	Advanced nurse inserter 49% Medical doctor 35% General nurse 5%	2% CHG in 70% IPA (Solu-Prep, 3 M, St Paul) Insyte Autoguard BC (BD Medical, Sandy) Connecta Extension tubing with Smart-Site Needle-Free Valves (BD) Sorbaview SHIELD-SV233, (Centurion Medical Products, Williamston)	72–96 h removal
Corley [31] RCT, *N* = 104	Adults Medical 29% Surgical emergent 24% Surgical elective 44% Trauma 3% Infection at recruitment 29% Wound at recruitment 38% Comorbidities 91% Overweight/Obese 70%	Multiple attempts 21% Hand 3% Wrist 15% Forearm 82%	Advanced nurse inserter 100%	Tegaderm IV Transparent Film Dressing with Border 1635 (10.5 × 8.5 cm), and two strips of sterile Medipore H Soft Cloth Surgical Tape on the extension tubing (both 3 M, St Paul) 70 patients had two additional sterile Medipore tape strips over the PIVC hub. 34 patients had additional tubular bandage (Tubifast, Molnlycke Health Care, Gothenburg)	72–96 h removal

*RCT*  Randomised controlled trial, *ED*  Emergency Department, *IPA*  IIopropyl alcohol, *CHG*  Clorhexidine gluconate, *IV*  Intravenous, *Prep*  Preparation

### PIVC infections—primary and secondary endpoints

Of 14,606 PIVCs (50,096 device-days), there were five local infections (0.034% [95% CI 0.011–0.080] of catheters; 0.100 [95% CI 0.042–0.240] per 1,000 device-days) (Table 2). As per the definition, patients with local infections did not have BSIs. Six (0.041% [95% CI 0.015–0.089]) patients developed a PIVC-associated BSI (0.120 [95% CI 0.054–0.267] per 1000 device-days).

SAB incidence was 0.007% (95% CI 0.0002–0.038) or 0.020 (0.003–0.142) per 1,000 device-days. Of the 6 PIVC-associated BSI cases, four also met the stricter criteria for PIVC-related BSI (0.027% [0.007–0.070]; 0.080 [0.030–0.213] per 1000 device-days).

**Table 2:** PIVC local and bloodstream infections, micro-organisms, and day of dwell in 14,606 PIVCs

	Local infection (CVS-VASC) *N* = 5		PIVC-associated BSI (primary BSI) *N* = 6	
	Organism	PIVC dwell	Organism	PIVC dwell
Van Donk [15] (*N* = 316)	0		0	
Rickard [16] (*N* = 603)	0		0	
Rickard [17] (*N* = 5907)	*n* = 1		*n* = 2	
	Purulence; no growth	Day 9 (201 h)	*S. aureus*	Day 4 (94 h)
			*E. cloacae*	Day 6 (140 h)
Marsh [18] (*N* = 85)	0		0	
Bugden [19](*N* = 369)	0		0	
Keogh [20] (*N* = 160)	0		0	
Marsh [25], (*N* = 1578)	0		0	
Marsh [23] (*N* = 119)	0		0	
Marsh [24] (*N* = 300)	0		0	
Rickard [22] (*N* = 1709)	*n* = 1		*n* = 3	
	*S. aureus*	Day 3 (48 h)	*E. cloacae/ Citrobacter braakii*	Day 2 (44 h)
			*E. cloacae*	Day 2 (33 h)
			*Pseudomonas aeruginosa*	Day 7 (150 h)
Kleidon [26] (*N* = 319)	*n* = 1 Purulence; no growth	Day 4 (93 h)		
Larsen [29] (*N* = 396)	*n* = 2 *E. cloacae* Purulence; no growth	Day 4 (77 h) Day 1 (24 h)	*n* = 1 *Proteus mirabilis*	Day 5 (102 h)
Keogh [28] (*N* = 619)	0		0	
Kleidon [27] (*N* = 55)	0		0	
Rickard [30] (*N* = 1759)	0		0	
Marsh [32] (*N* = 200)	0		0	
Marsh [34] (*N* = 69)	0		0	
Corley [31] (*N* = 104)	0		0	

*PIVC* Peripheral intravenous catheter, *CVS-VASC* Cardiovascular system—vascular infection, *S.* Staphylococcus; *E.* Enterobacter, *h* Hours

### Primary BSI case characteristics

Characteristics of the six PIVC-associated BSIs are presented in Table 3. The predominant BSI organism was *Enterobacter cloacae*, which occurred in three patients (including one in conjunction with *Citrobacter braakii*), with one case each of *Proteus mirabilis*,* Pseudomonas aeruginosa* and *S. aureus.* Patients were all adults, aged 53–71 years, with varied medical (cancer, vascular, or cardiac), and surgical (two abdominal; one orthopaedic) primary diagnoses. Five of the six patients were male, aged > 60 years, and/or had multiple previous PIVCs during the admission. Three patients had a co-existing abdominal drain. Five cases occurred while the PIVC was in situ, and one occurred within 24 h after removal. PIVC insertion site assessments were abnormal on the day of removal in five patients, with one patient developing symptoms on the day after removal. The most common abnormality at removal was pain or tenderness, rated on a 0–10 (no pain to most severe pain) scale by patients as 0 (*n* = 1), 1–2 (*n* = 2), 2–5 (*n* = 2), and 5 (*n* = 1).

Five patients had their PIVC removed in response to the BSI or a symptomatic insertion site. The remaining patient had the PIVC left in place for a further two days after the blood culture was drawn as the PIVC was not the suspected cause of infection. PIVC removal appeared delayed in four patients who were all difficult to cannulate and had already had multiple devices, in the context of few advanced inserter staff with ultrasound skills and possible patient refusal of routine removal. Two patients had idle PIVCs (for 2 and 6 days respectively). No metastatic infection complications were observed, and all patients responded to intravenous antibiotic therapy, with one remaining on oral antibiotics at hospital discharge.

**Table 3:** Case details for six PIVC-associated BSIs in 14,606 PIVCs

Case	Sex	Age (> 60 yrs)	Biliary stent/GI surgery	Cancer diagnosis^a^	PIVC site complications	Forearm location	PIVC idle > 24 h	PIVC dwell time	Organism/s	Other queried BSI sources	Comments
1^b^	Male	Yes	No	Yes	Erythema, tracking	Yes	No	140 h	*E. cloacae*; blood and PIVC tip	Urinary; GI blockage	PIVC not replaced routinely (despite policy)
2^b^	Female	No	Yes; co-existing drain	No	Tenderness	Yes	No	33 h	*E. cloacae*; blood and PIVC tip	No	Co-existing PIVC
3^b^	Male	Yes	No	No	Tenderness, erythema < 1 cm, swelling 1–2.5 cm	Yes	Yes	150 h	*P. aeruginosa*; blood, PIVC tip, PIVC site swab	No	PIVC not replaced routinely (despite policy)
4^c^	Male	Yes	Yes; co-existing drain	Yes	Tenderness, pain	Hand	No	94 h	*S. aureus*; blood.	Biliary drain	New fever (39.4 °C) immediately after biliary drain removal
5	Male	Yes	Yes; co-existing drain	Yes	Tenderness, small palpable cord	Yes	Yes	44 h	*E. cloacae*, *Citrobacter braakii*; blood	Biliary leak	Recent BSI (matching organism); event outside repeat infection period
6^b^	–	–	–	Yes	Tenderness (3/10), erythema (1 cm), purulence	–	–	102 h	*Proteus mirabilis*; blood	–	PIVC not replaced routinely (despite policy). Febrile, delirious. Recent *S. aureus* local infection in another PIVC.

^a^admitting diagnosis or comorbidity; ^b^meets PIVC related BSI criteria; ^c^meets S. aureus BSI criteria; *GI* Gastrointestinal, – unknown (limited dataset available)

### Daily risk of PIVC-associated BSI and modelling of the hazard function

**Fig. 1:**
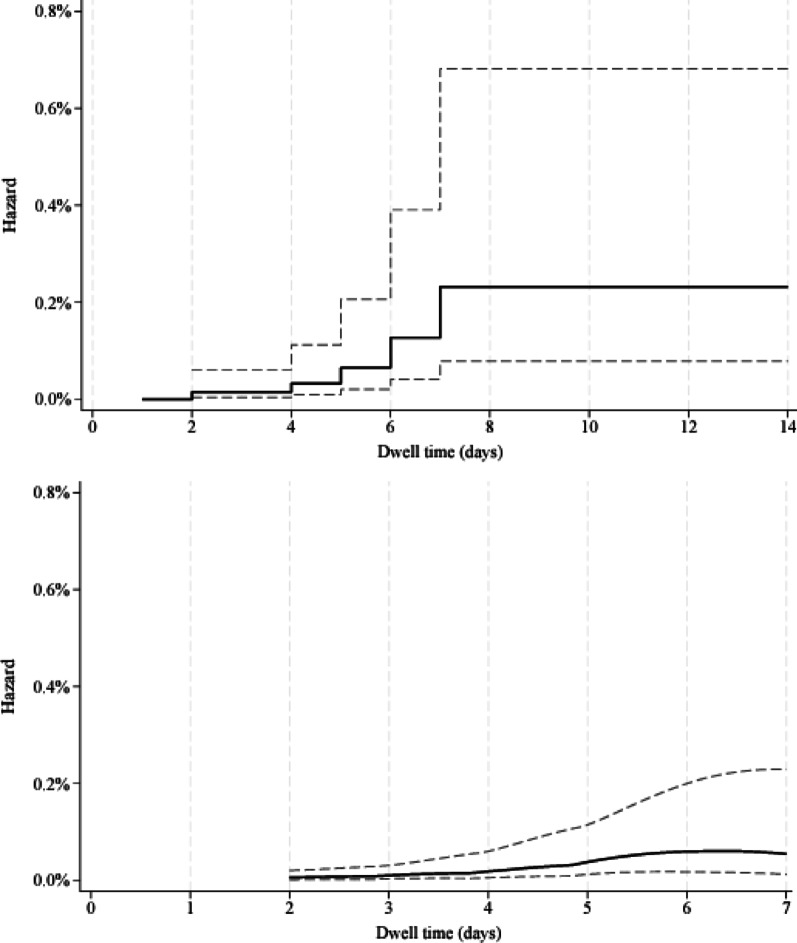
**a** (top) Nelson-Aalen hazard function (incl. 95% confidence interval [CI]) of PIVC-associated BSI over time. **b** (bottom). Smoothed hazard estimate (incl. 95% CI) of PIVC-associated BSI over time. **a** and **b** solid lines are estimates; dashed lines are 95% CI

PIVC dwell ranged from < 1–42 days (Table 4). PIVCs were proportionally removed on: day 1 (day of insertion; 10%), day 2 (26%), day 3 (25%), day 4 (18%), day 5 (10%), day 6 (5%), day 7 (3%), day 8–14 (3%), and ≤ 1% used for 3–6 weeks. The six PIVC-associated BSIs occurred on dwell days 2 to 7 (Table 4). The highest observed hazard was 0.10% (95% CI 0.00–0.39) on day 7, and the cumulative failure probability plateaued at 0.23% (95% CI 0.08–0.68) by day 7. The cumulative hazard and smoothed hazard are shown on Fig. 1a and b, respectively. The BIC value of the model assuming exponential distribution was lower by 3.6 than for the Weibull distribution with a shape parameter of *p* = 2.1 (95% CI 1.4–3.3) indicating a constant hazard over time.

**Table 4:** Life table of PIVC per-day survival from PIVC-associated BSI in 14,606 PIVCs

Day of dwell	Starting number	PIVC-associated BSI	Removed	Hazard (95% CI)
1	14,606	0	1494	0.00%
2	13,112	2	3758	0.02% (0.00–0.04)
3	9352	0	3636	0.00%
4	5716	1	2629	0.02% (0.00–0.06)
5	3086	1	1469	0.03% (0.00–0.12)
6	1616	1	660	0.06% (0.00–0.23)
7	955	1	410	0.10% (0.00–0.39)
8	544	0	235	0.00%
9	309	0	109	0.00%
10	200	0	74	0.00%
11	126	0	39	0.00%
12	87	0	29	0.00%
13	58	0	9	0.00%
14	49	0	11	0.00%
15	38	0	13	0.00%
16	25	0	3	0.00%
17	22	0	3	0.00%
18	19	0	5	0.00%
19	14	0	5	0.00%
20	9	0	1	0.00%
31	8	0	1	0.00%
36	7	0	2	0.00%
42	5	0	5	0.00%

Dwell = number in place at commencement of that interval day (days with no devices removed, e.g., Day 33 are not shown); removed = devices removed that day; CI values cannot be calculated when the point estimate is zero

## Discussion

Our meta-synthesis of 18 prospective studies and 14,606 PIVCs identified PIVC-associated BSI incidence at one in 2434 PIVCs (0.041%; 0.12/1000 device-days). This was slightly higher than the incidence of 0.028% reported in a meta-analysis of 38 predominantly prospective studies with clearly defined PIVC-associated BSI [8], which may reflect our rigorous approach to follow up or that Australian burden is slightly higher than global rates. In contrast, our PIVC-associated BSI incidence was lower than the 0.18% “PIVC-related BSI” (also without requirement for microbiological confirmation of PIVC as the source) in a review of 37 studies published from 1980 to 2015, suggesting that risk has reduced over time, or reflecting the varied data sources (prospective studies cf. surveillance databases) [5]. In resource-limited countries far higher incidence has been reported with “PIVC related-BSI” (also without requirement for microbiological confirmation of PIVC as the source) at 2.41/1,000 PIVC-days (149,609 ICU patients; 743,508 PIVC-days) [38], indicating both a higher risk in the critically ill and a disproportionate burden of disease in developing economies where personnel and technical resources are limited. PIVC-associated BSI is a surveillance definition and are not all are truly causally related. When we restricted our results to the diagnostic definition of PIVC-*related* BSI, incidence dropped to 0.027% (one in 3,652 PIVCs). This is lower than the 0.1% PRBSI reported two decades ago in a systematic review of 10,910 prospectively studied PIVCs [9]. The difference in results may be explained by practice and product evolution over time, e.g., catheter materials technologies such as needleless connectors, and the addition of chlorhexidine gluconate to alcohol-based skin decontamination prior to insertion.

*S. aureus* is commonly implicated in PIVC-associated BSI [1, 2, 5], but we found only a single case, reflecting an incidence of 0.007% PIVCs, *and E. cloacae* was predominant (three cases of the six cases; 0.02% of PIVCs, including one co-infection with *C. braakii*). Gram-negative organisms may now be the predominant source of PIVC-associated BSI in Australia, as has been reported in other nations [3, 38]. Lengthy hospitalisation and complex comorbidities may have influenced these findings. However, BSI attribution to the PIVC was rarely straightforward; three cases were suspected by treating clinicians to be secondary to complicated gastrointestinal surgery (including one with observed intraoperative purulence), but without microbiological evidence of a gastrointestinal source patients still technically met the PIVC-associated BSI criteria. Three PIVC-associated BSI patients met the additional CRBSI criteria (increased likelihood of true attributability), yet even of these, one appeared to have haematogenously seeded from the clinically suspected primary gastrointestinal source. Just as CLABSI definitions were revised to account for mucosal barrier injury infections [35], similar consideration may be needed for PIVC-associated BSI definitions.

We observed local infections at one in 2921 PIVCs (0.034% of catheters; 0.100/1000 device-days). A systematic review reported a higher incidence of 0.150% (30 studies; 0.651/1000 device-days) [8], which may reflect our criteria requiring no associated BSI. Microbiological investigation of symptomatic PIVC sites is not always performed in practice, which also lowers rates of detection. Half of our PIVC-associated BSI cases also had positive local infection criteria, demonstrating the importance of inspection of the insertion site for complications and early removal in response. A similar pattern was seen in 62 retrospective PIVC-associated BSI cases from Japan, in whom 63% had phlebitis, and for those with bacterial cellulitis, mortality was significantly higher [2]. We note that of the local infection patients who had exudate sent for microbiological culture using the roll-plate technique, only one yielded a result, suggesting a need for superior diagnostic techniques.

Our insights into the clinical circumstances surrounding PIVC-associated BSI include that all cases occurred in large metropolitan centres, not smaller regional hospitals, emphasising the importance of risk adjustment in cross-institutional comparisons. Older age and comorbidities were common, consistent with earlier work [1, 3, 39] and we saw no infections in paediatrics. We found gastrointestinal procedures and cancer diagnoses to predominate, suggesting priority groups for prevention and surveillance could be those with complex and lengthy admissions. Three patients had co-existent wound drains; a novel finding requiring further research and may explain the preponderance of gram-negative organisms. All cases had multiple PIVCs during admission including many delayed and difficult insertions due to limited suitable veins and poor availability of advanced nurse inserters who can improve insertion success [23]. Post-insertion, infected PIVCs were commonly not removed despite insertion site complications and/or idle status, emphasising the need for decision support, e.g., the I-DECIDED^®^ tool [40]. A recent secondary analysis of three RCTs from France identified insertion over a joint (wrist/antecubital fossa) to be significantly associated with infection risk [11]. We did not see this pattern, perhaps due to fewer of these PIVC sites in our studies which typically excluded planned short-dwell PIVCs (such as those inserted in emergency or anaesthetic departments).

We present the first life table analysis and hazard function modelling of the PIVC *per-day risk* of primary BSI, finding a constant risk over time, with greater certainty over the first five days of dwell, where rates were almost identical. The average PIVC dwell was 3.4 days. Each day of PIVC therapy carries risk, and biofilm can develop in PIVCs during dwell which supports the concept of a ‘tipping point’ where risk from biofilm may exceed risk of a new insertion. However, our work supports that, at least for the first 5 days, there is no benefit in routinely replacing PIVCs, distributing this between multiple PIVCs does not benefit the patient overall. Ensuring clinical staff knowledge and action regarding appropriate PIVC removal criteria remains vital. Previous studies using multivariable analyses have also not observed longer per-PIVC dwell to be significantly associated with infection [3, 12, 39]. Analysis of surveillance databases is more likely to attribute longer per-PIVC dwell time as a factor associated with PIVC-associated BSIs [5]; this may reflect longer *overall* IV therapy in more highly acute and thus infection-prone patients which is a known risk for infection [1]. Such patients also commonly have limited suitable peripheral veins, leading to difficult insertions with suboptimal technique, and reluctance by staff to remove PIVCs even if idle or symptomatic, knowing replacement may be challenging without access to vascular access specialists. Historical reliance on routine removal for infection prevention has perhaps surreptitiously reduced attention on risk-stratification, improving insertion quality, and vigilance in the early days of dwell (e.g., two PIVC-associated BSI cases in our series occurred on Day 2). While a Cochrane review (7 RCTs; 7323 patients) reported very low PIVC-associated BSI incidence regardless of removal policy (0.053% PIVCs routine; 0.028% PIVCs clinically indicated) [41], institutions adopting clinically indicated removal have reported both decreased and increased infections [42, 43], emphasising the importance of new systems that explicitly support clinical staff to recognise and enact appropriate removal opportunities [40].

Our results reflect Australian healthcare and may not be generalizable to other nations, particularly those with resource limitations or where PIVCs are extensively used in ICUs. Many primary studies excluded patients with very short-dwell PIVCs, and infection rates would have been even lower if these were included. Rates may also have been reduced by patients being enrolled in research studies, however the researchers and research nurses did not deliver care, and most studies were not specifically focussed on infection prevention. Standards of insertion and maintenance were not optimal, with contemporaneous work in Australia showing 43% multiple-attempt insertions, 25% suboptimal dressings, and 23% of PIVCs idle [7], thus our results reflect “real world” (not ideal) conditions. We were only able to examine the risk factors available to us (including one case with a limited dataset) and could not examine the independent risk factors identified in a Japanese cohort [3] (showering, Pitt bacteraemia score ≥ 2, absence of parenteral nutrition). Because infection incidence was low, we were unable to explore sub-groups, such as paediatrics. We had little data on PIVCs dwelling >7 days, thus our estimates are imprecise after this time, and further research is needed to understand risk with very prolonged use. Study strengths included prospective recruitment at PIVC insertion, the large sample size, data collection by research nurses, and blinded outcome assessments using NHSN criteria. Generalisability was strengthened by including PIVCs across clinical settings, predominantly inserted and cared for by junior medical and general nursing staff, with a smaller number placed by specialist IV nurses.

Although we found infection risk per PIVC to be very low, infection incidence across the health system is serious due to the substantial numbers used. In Australia (population approximately 27 million in 2024), 12.3 million PIVCs are purchased by the public health system annually, of which perhaps 25% are used for failed insertions and another 15% are removed the same day without infection [44]. Our data indicate with 95% confidence that 1176 to 6976 PIVC-associated BSIs occur in Australia alone each year. These results will be useful for future benchmarking and to identify areas for improvement. We had no reports of manufacturer-contaminated products (e.g., fluids) over the period, thus poor insertion or post-insertion care were the likely source of most infections (haematogenous seeding is also possible).

PIVC infections can be avoided through improved insertion, maintenance and decision-making about timely removal, as outlined in the Australian PIVC Clinical Care Standards WHO Guidelines [13, 14] and the Infusion Nursing Society Standards [45]. Additional measures could also be targeted at high-risk groups, namely males, those over 60 years, those with difficult intravenous access, multiple comorbidities, cancer diagnoses or invasive gastrointestinal procedures. Better insertion, including expanded access to advanced inserters, combined with standardised, post-insertion decision-support to prompt early appropriate removal, could see PIVC infections reduced close to zero.

## Supplementary Information

Below is the link to the electronic supplementary material.

Supplementary Material 1.

## Data Availability

Participants did not consent to re-use of their data for all primary studies and no participants consented to public availability of their data. Anonymous data from some studies where participants did consent to future use will be available on submission of a satisfactory protocol and reasonable request to the corresponding author, and human research ethics approval from University of Queensland.
