# Supplementary material for: Infection risk of peripheral intravenous catheters: meta-synthesis of 18 prospective studies with 14,606 catheters
Source: Antimicrob Resist Infect Control. 2025 Oct 27;14:129. doi: 10.1186/s13756-025-01645-z (PMC12560543; doi:10.1186/s13756-025-01645-z)
Supplement: Supplementary file 1 — Supplementary Material 1. [file 13756_2025_1645_MOESM1_ESM.docx]

# Supplementary materials

## Primary outcome definitions

*Local infection*: NHSN i.e., *Cardiovascular System VASC-Arterial or Venous Infection* (CVS-VASC) adult criteria,[35] no laboratory-confirmed BSI but at least one of the following criteria:

1. Organism(s) from extracted vein identified by culture-based/non-culture-based testing, performed for clinical diagnosis (not surveillance)
2. Evidence of venous infection on gross anatomic or histopathologic exam
3. At least one of: fever (>38.0 °C), pain with no other recognized cause, erythema with no other recognized cause, or heat with no other recognized cause at the vascular site involved, AND more than 15 colony forming units (cfu) cultured from PIVC tip using semi-quantitative method
4. Purulent drainage from the PIVC insertion site.

*Primary BSI*: NHSN laboratory-confirmed bloodstream infection (LCBI) definition,[35] in summary:

- a bacterial or fungal pathogen isolated from blood cultures, and not related to infection at another site, or
- two positive sets of blood cultures and clinical features of infection required for common commensal organisms.
